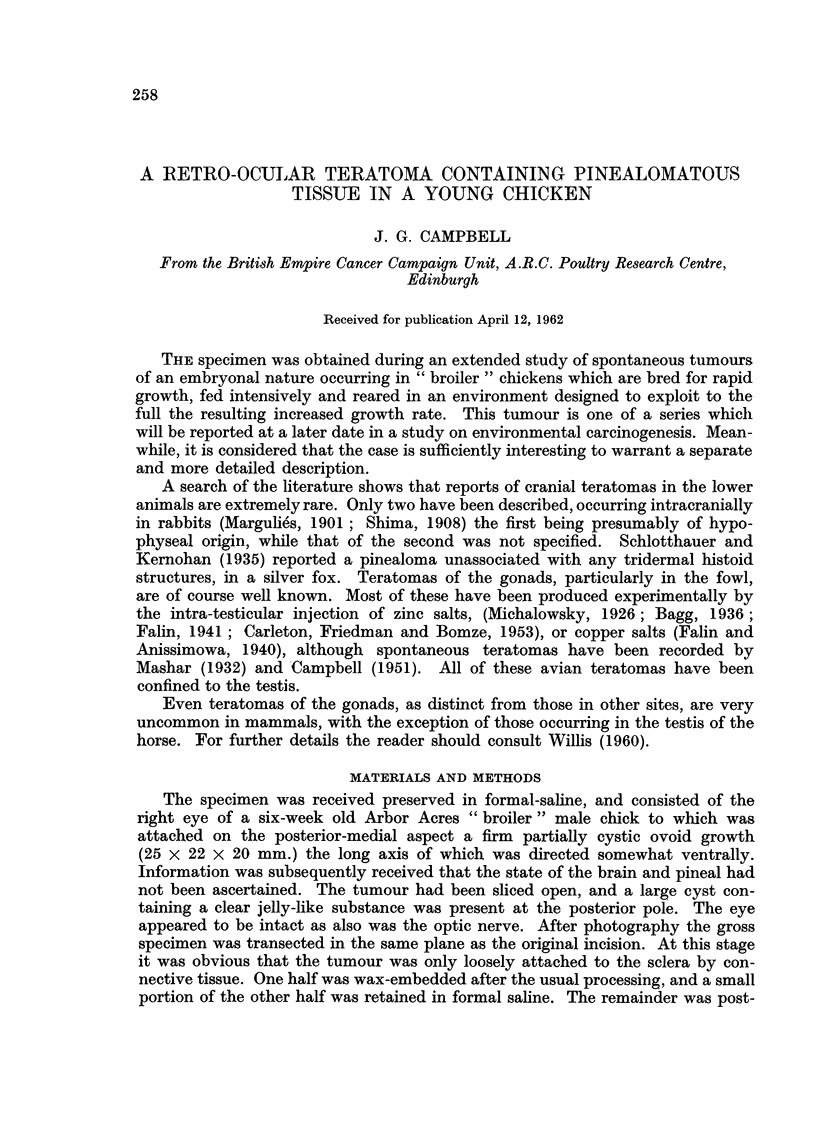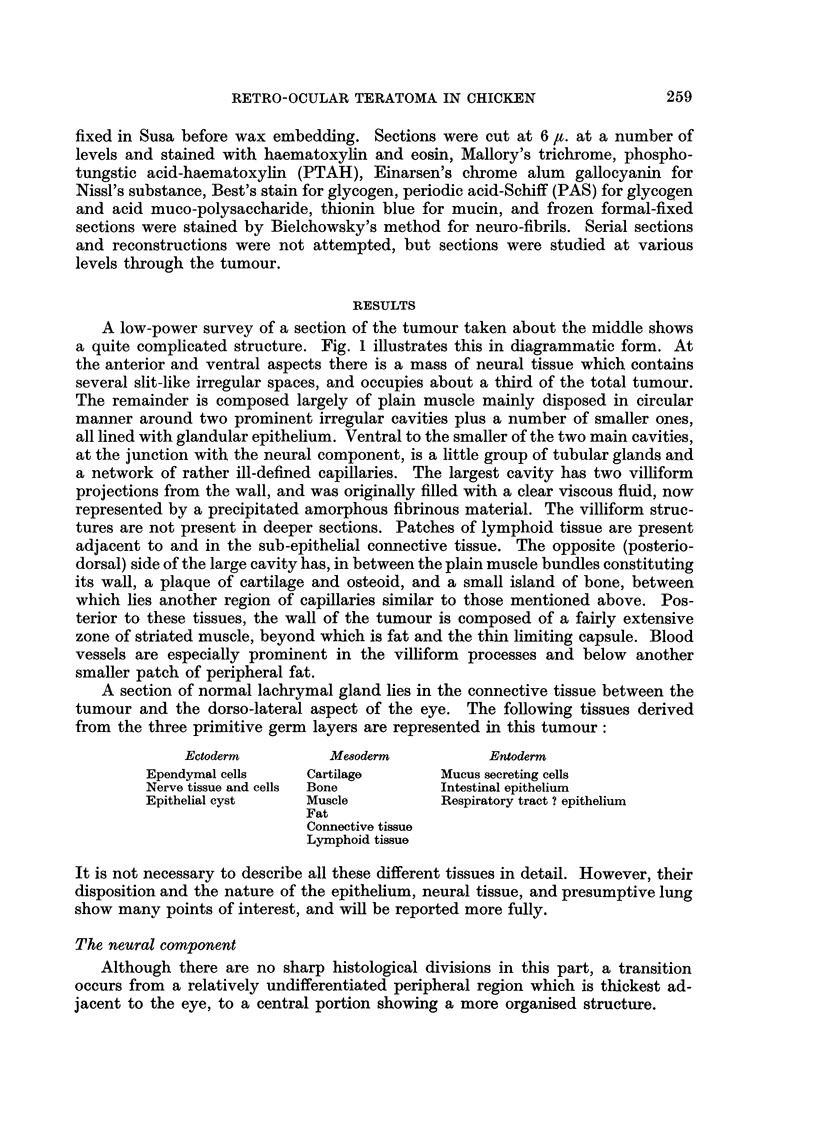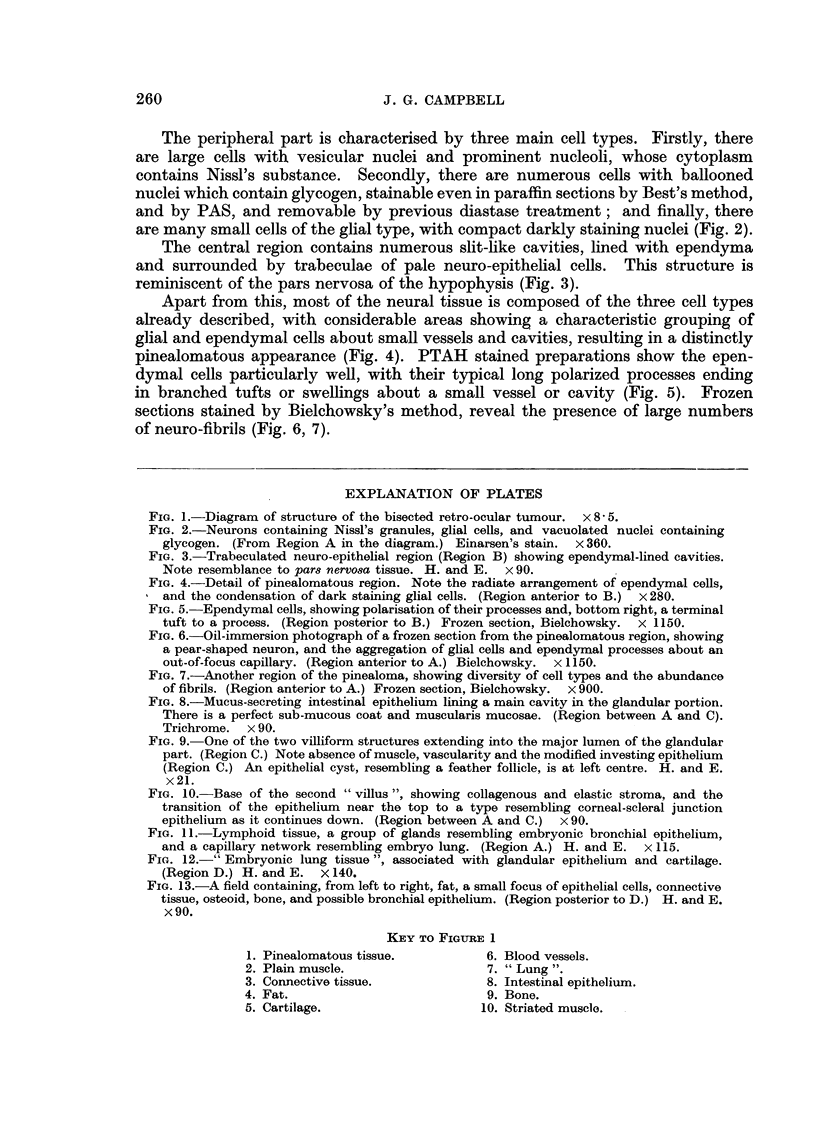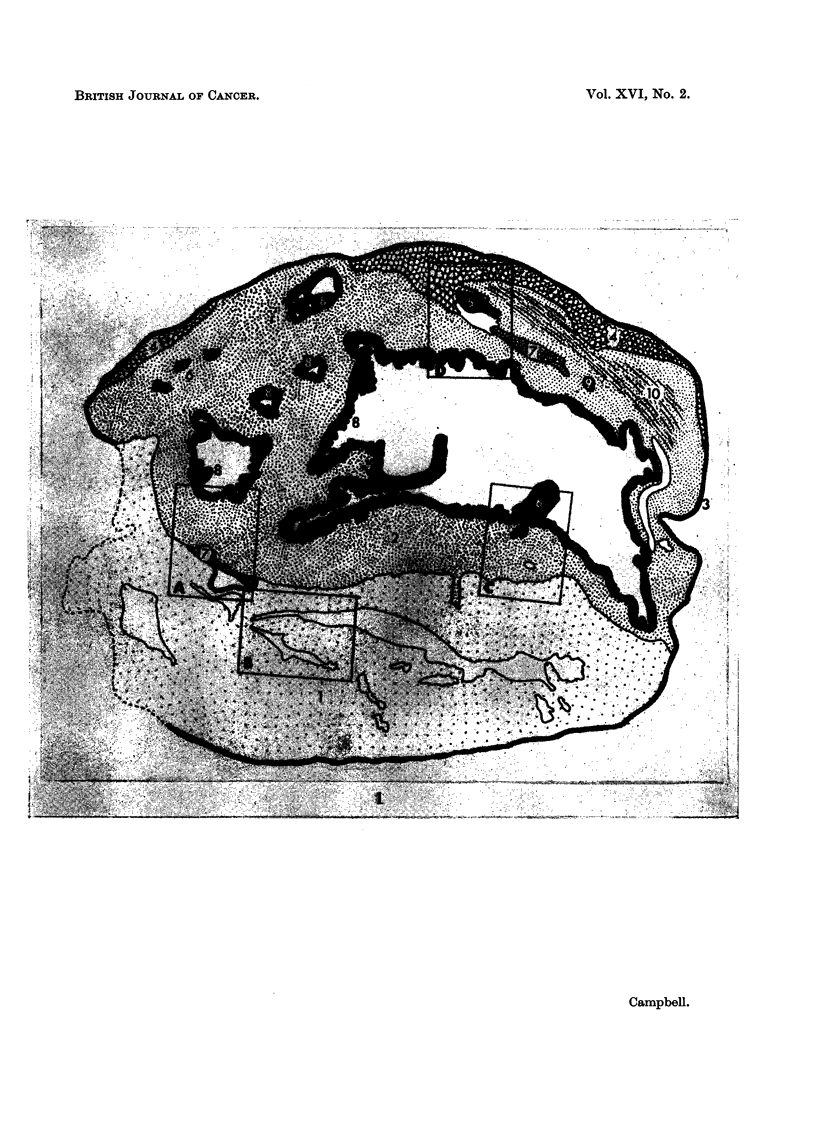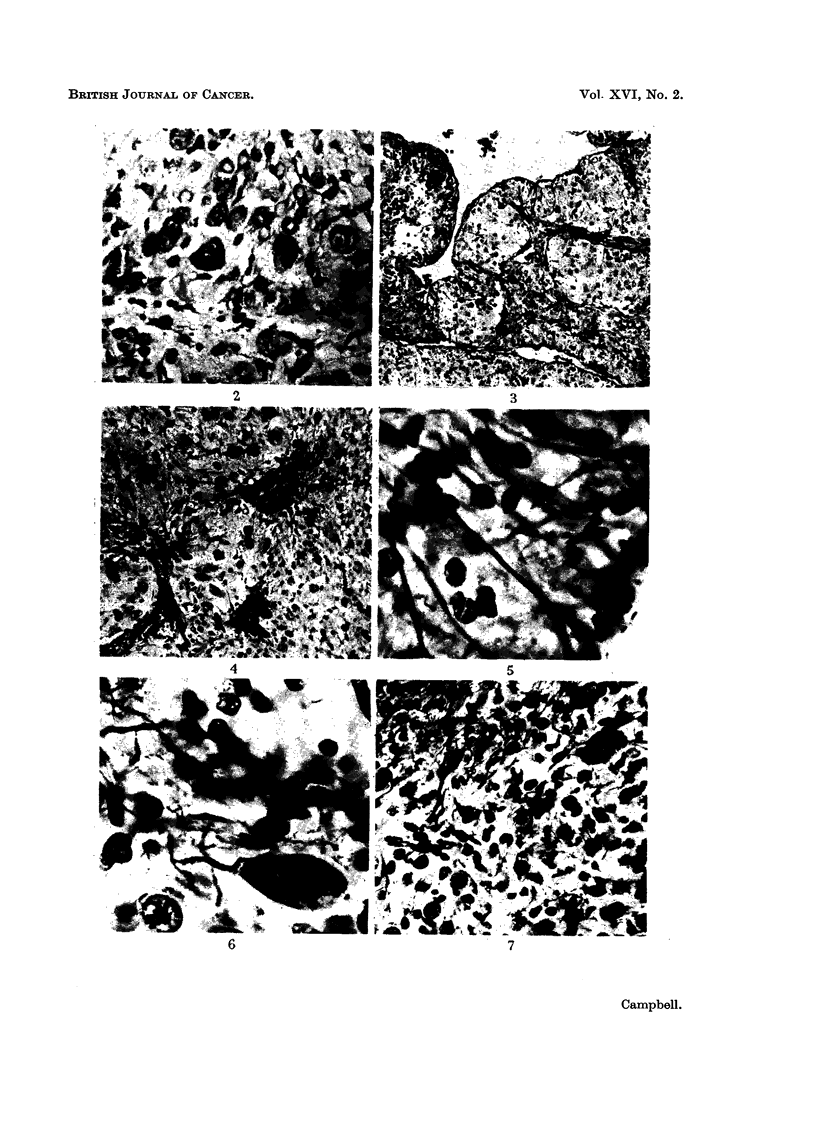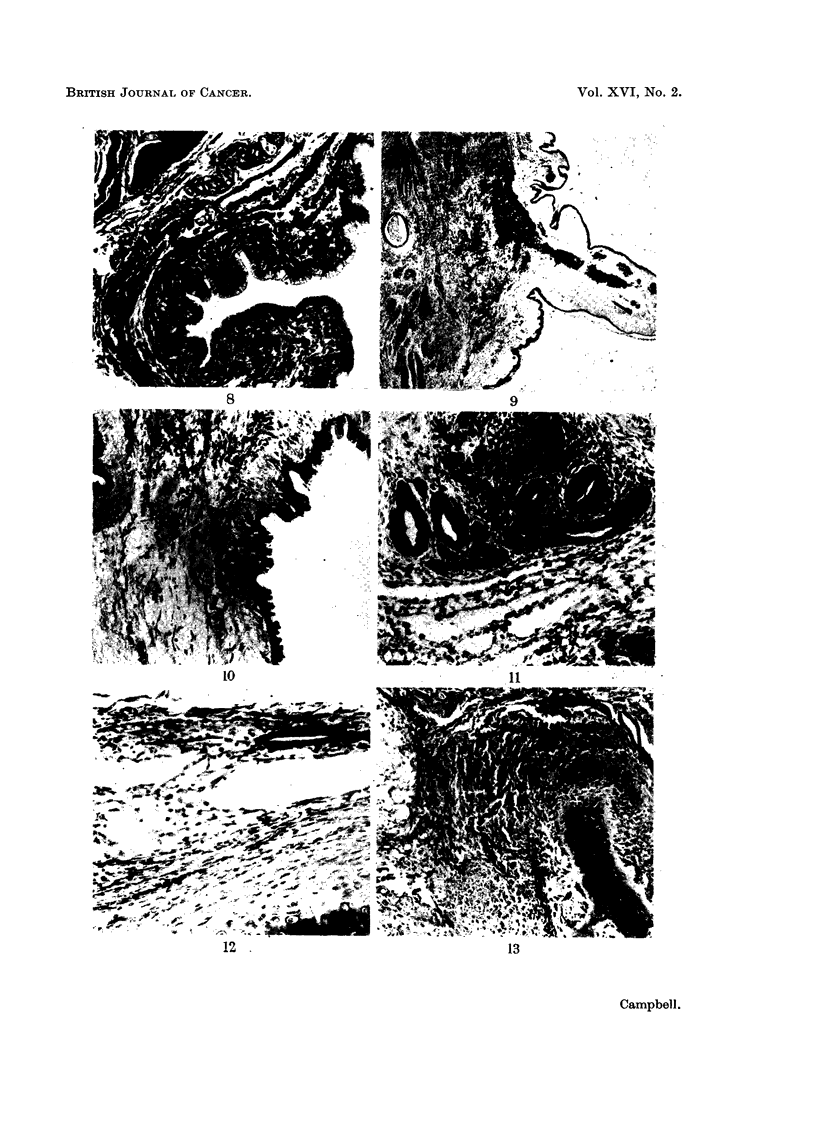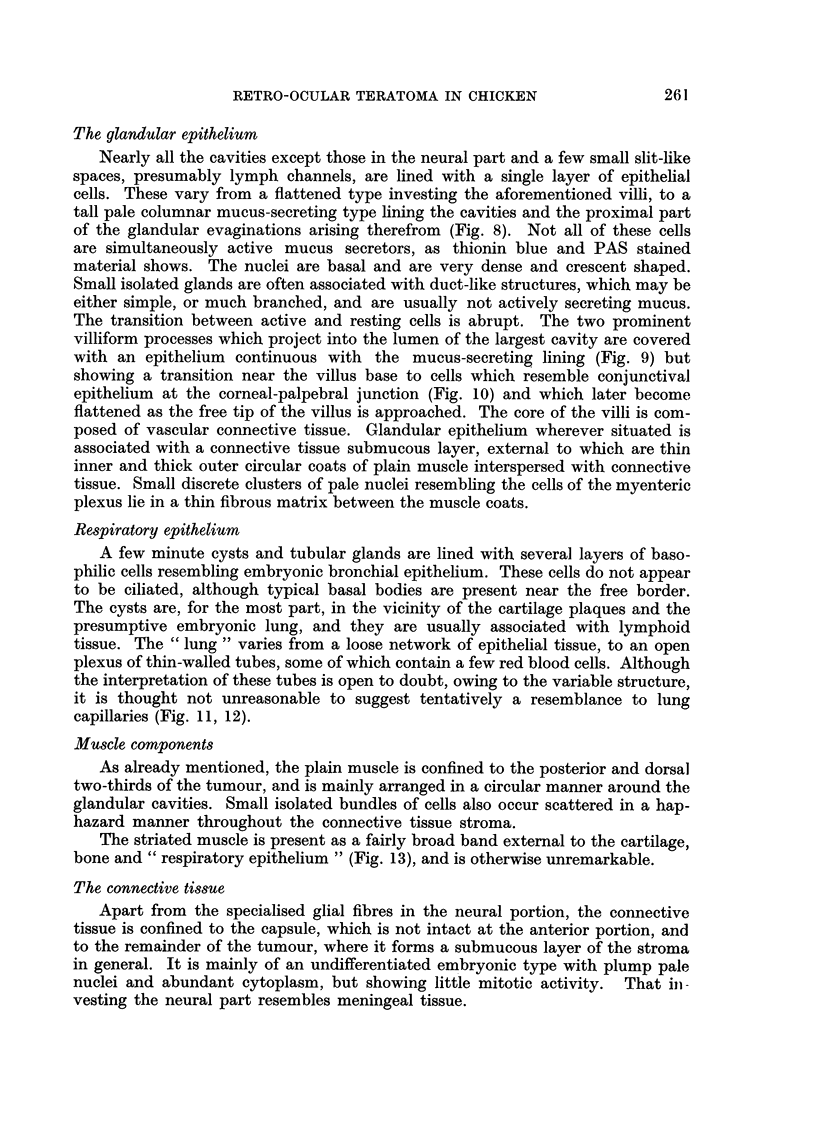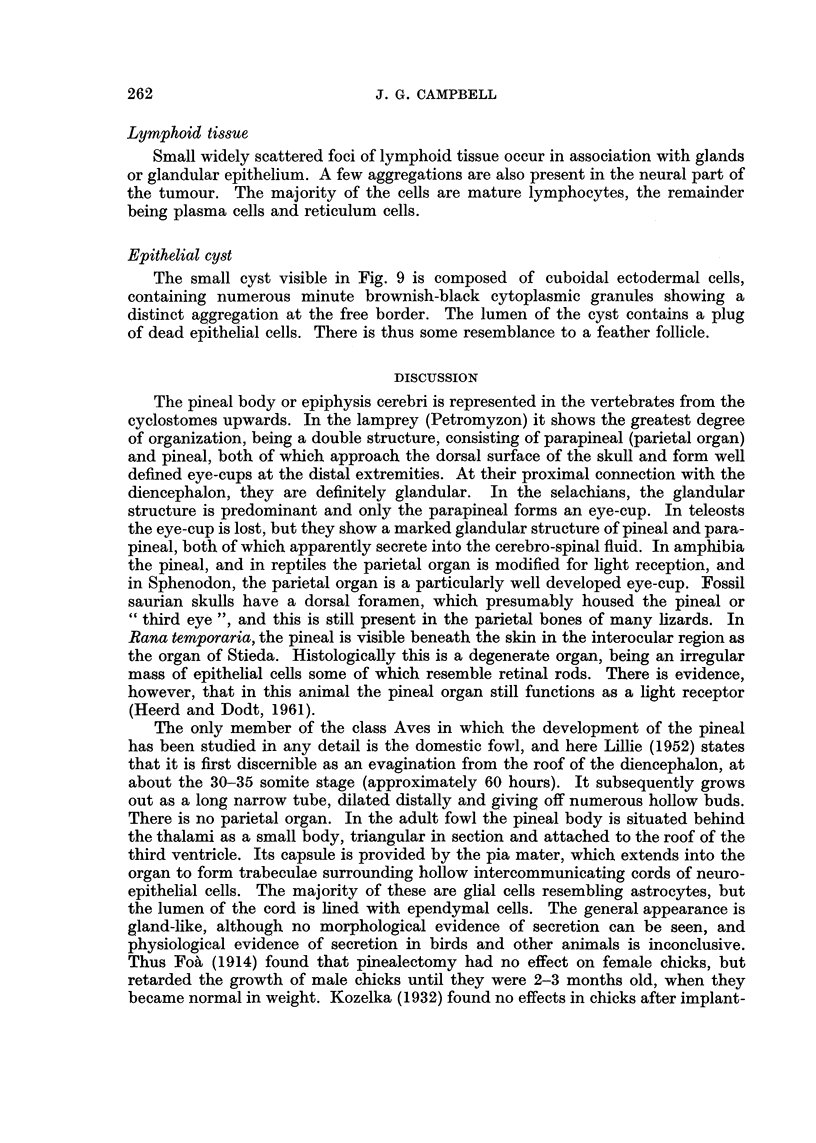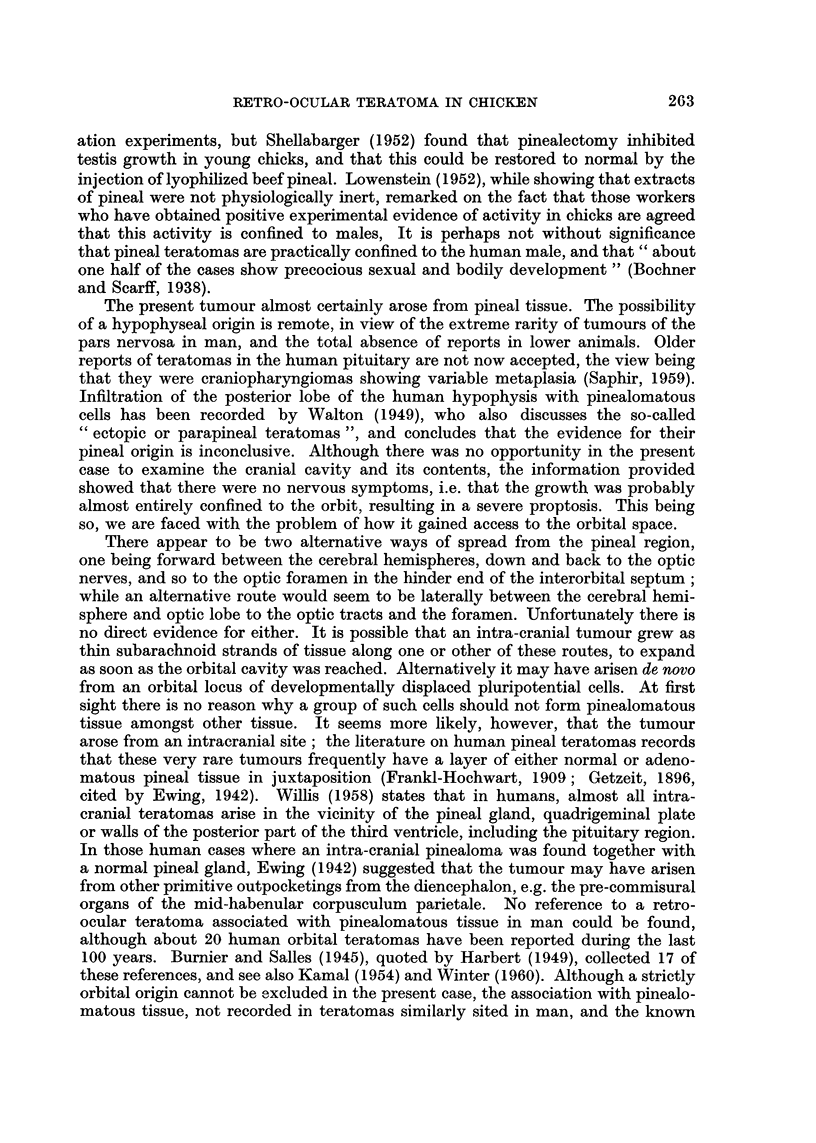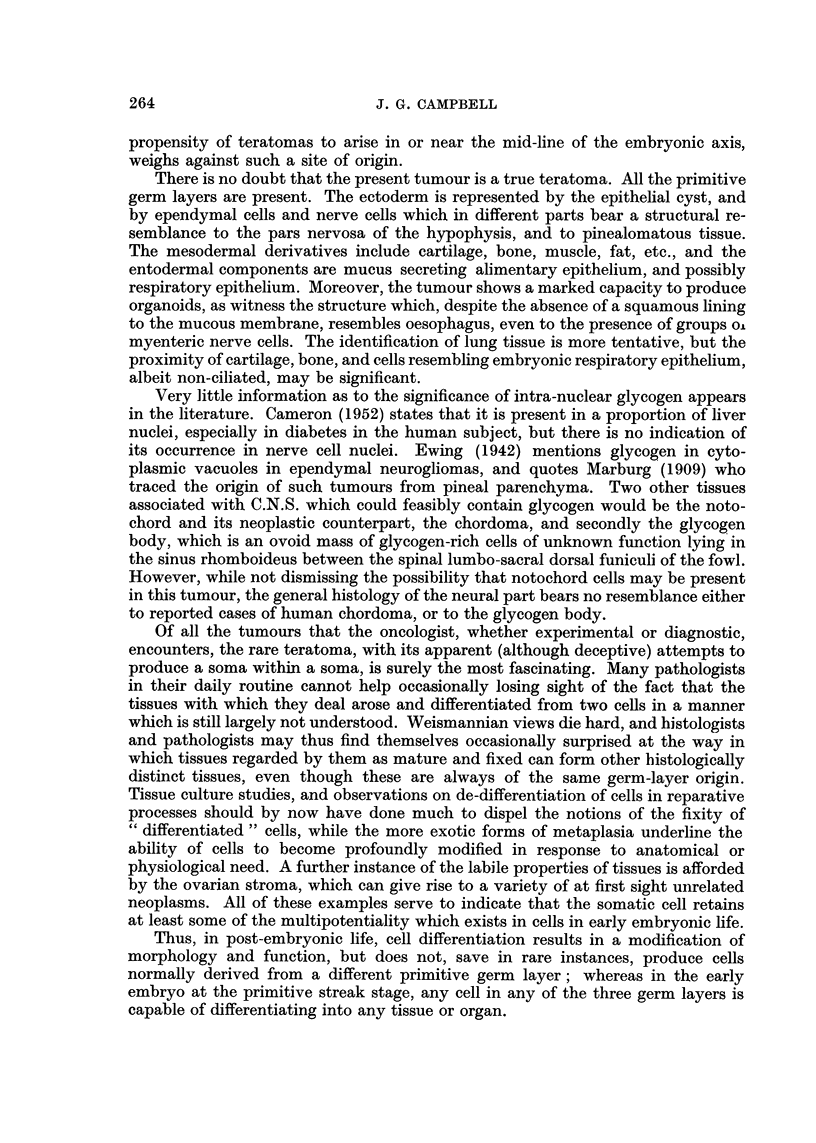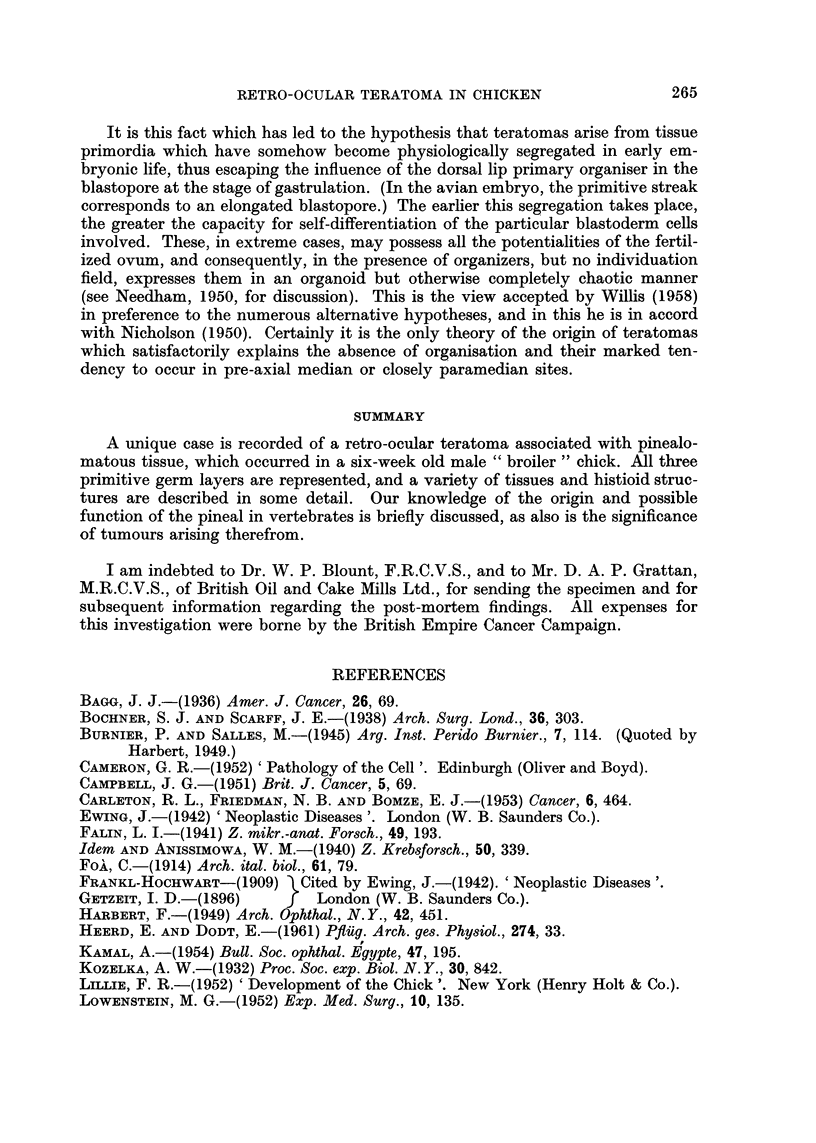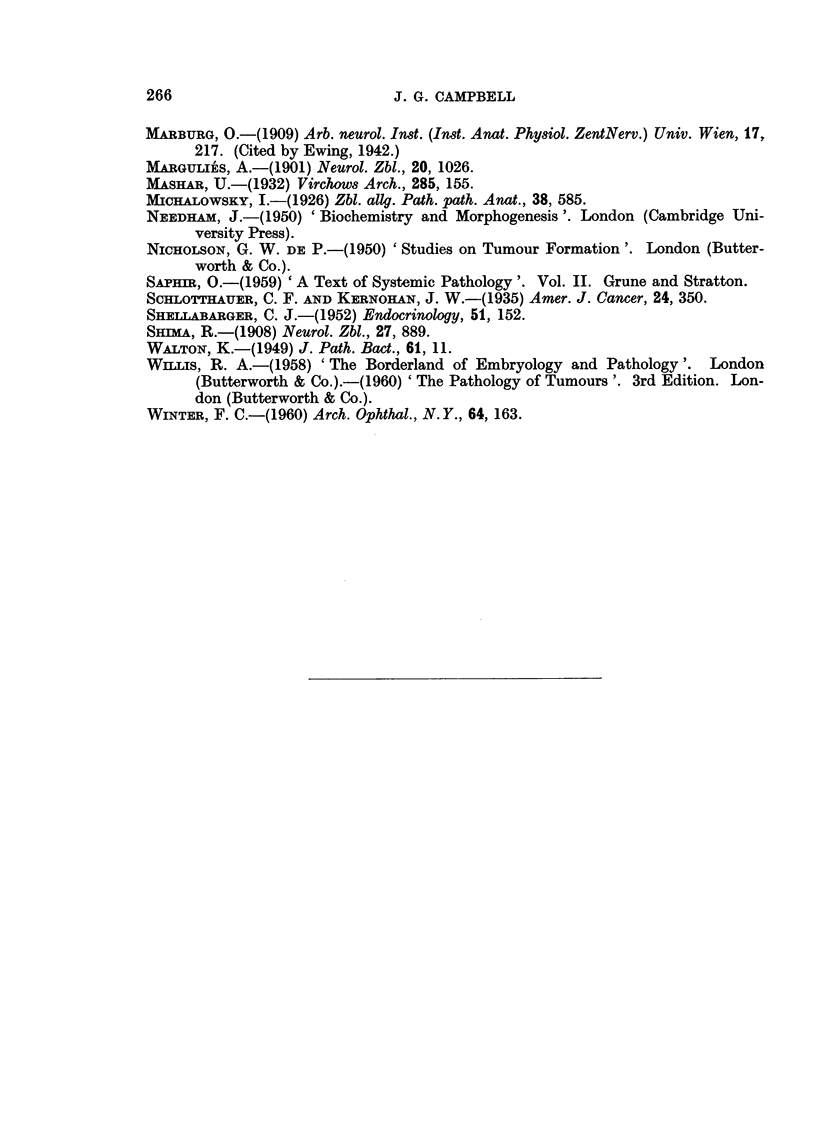# A Retro-Ocular Teratoma Containing Pinealomatous Tissue in a Young Chicken

**DOI:** 10.1038/bjc.1962.30

**Published:** 1962-06

**Authors:** J. G. Campbell

## Abstract

**Images:**


### A RETRO-OCULAR TERATOMA CONTAINING PINEALOMATOUS TISSUE IN A YOUNG CHICKEN

J. G. CAMPBELL

*From the British Empire Cancer Campaign Unit, A.R.C. Poultry Research Centre, Edinburgh*

Received for publication April 12, 1962

THE specimen was obtained during an extended study of spontaneous tumours of an embryonal nature occurring in " broiler " chickens which are bred for rapid growth, fed intensively and reared in an environment designed to exploit to the full the resulting increased growth rate. This tumour is one of a series which will be reported at a later date in a study on environmental carcinogenesis. Meanwhile, it is considered that the case is sufficiently interesting to warrant a separate and more detailed description.

A search of the literature shows that reports of cranial teratomas in the lower animals are extremely rare. Only two have been described, occurring intracranially in rabbits (Marguliés, 1901 ; Shima, 1908) the first being presumably of hypophyseal origin, while that of the second was not specified. Schlotthauer and Kernohan (1935) reported a pinealoma unassociated with any tridermal histoid structures, in a silver fox. Teratomas of the gonads, particularly in the fowl, are of course well known. Most of these have been produced experimentally by the intra-testicular injection of zinc salts, (Michalowsky, 1926 ; Bagg, 1936 ; Falin, 1941 ; Carleton, Friedman and Bomze, 1953), or copper salts (Falin and Anissimowa, 1940), although spontaneous teratomas have been recorded by Mashar (1932) and Campbell (1951). All of these avian teratomas have been confined to the testis.

Even teratomas of the gonads, as distinct from those in other sites, are very uncommon in mammals, with the exception of those occurring in the testis of the horse. For further details the reader should consult Willis (1960).

### MATERIALS AND METHODS

The specimen was received preserved in formal-saline, and consisted of the right eye of a six-week old Arbor Acres " broiler " male chick to which was attached on the posterior-medial aspect a firm partially cystic ovoid growth (25 × 22 × 20 mm.) the long axis of which was directed somewhat ventrally. Information was subsequently received that the state of the brain and pineal had not been ascertained. The tumour had been sliced open, and a large cyst containing a clear jelly-like substance was present at the posterior pole. The eye appeared to be intact as also was the optic nerve. After photography the gross specimen was transected in the same plane as the original incision. At this stage it was obvious that the tumour was only loosely attached to the sclera by connective tissue. One half was wax-embedded after the usual processing, and a small portion of the other half was retained in formal saline. The remainder was postfixed in Susa before wax embedding. Sections were cut at 6 $\mu$. at a number of levels and stained with haematoxylin and eosin, Mallory's trichrome, phosphotungstic acid-haematoxylin (PTAH), Einarsen's chrome alum gallocyanin for Nissl's substance, Best's stain for glycogen, periodic acid-Schiff (PAS) for glycogen and acid muco-polysaccharide, thionin blue for mucin, and frozen formal-fixed sections were stained by Bielchowsky's method for neuro-fibrils. Serial sections and reconstructions were not attempted, but sections were studied at various levels through the tumour.

### RESULTS

A low-power survey of a section of the tumour taken about the middle shows a quite complicated structure. Fig. 1 illustrates this in diagrammatic form. At the anterior and ventral aspects there is a mass of neural tissue which contains several slit-like irregular spaces, and occupies about a third of the total tumour. The remainder is composed largely of plain muscle mainly disposed in circular manner around two prominent irregular cavities plus a number of smaller ones, all lined with glandular epithelium. Ventral to the smaller of the two main cavities, at the junction with the neural component, is a little group of tubular glands and a network of rather ill-defined capillaries. The largest cavity has two villiform projections from the wall, and was originally filled with a clear viscous fluid, now represented by a precipitated amorphous fibrinous material. The villiform structures are not present in deeper sections. Patches of lymphoid tissue are present adjacent to and in the sub-epithelial connective tissue. The opposite (posterio-dorsal) side of the large cavity has, in between the plain muscle bundles constituting its wall, a plaque of cartilage and osteoid, and a small island of bone, between which lies another region of capillaries similar to those mentioned above. Posterior to these tissues, the wall of the tumour is composed of a fairly extensive zone of striated muscle, beyond which is fat and the thin limiting capsule. Blood vessels are especially prominent in the villiform processes and below another smaller patch of peripheral fat.

A section of normal lachrymal gland lies in the connective tissue between the tumour and the dorso-lateral aspect of the eye. The following tissues derived from the three primitive germ layers are represented in this tumour :

| *Ectoderm* | *Mesoderm* | *Entoderm* |
|---|---|---|
| Ependymal cells | Cartilage | Mucus secreting cells |
| Nerve tissue and cells | Bone | Intestinal epithelium |
| Epithelial cyst | Muscle | Respiratory tract ? epithelium |
| | Fat | |
| | Connective tissue | |
| | Lymphoid tissue | |

It is not necessary to describe all these different tissues in detail. However, their disposition and the nature of the epithelium, neural tissue, and presumptive lung show many points of interest, and will be reported more fully.

### *The neural component*

Although there are no sharp histological divisions in this part, a transition occurs from a relatively undifferentiated peripheral region which is thickest adjacent to the eye, to a central portion showing a more organised structure.

The peripheral part is characterised by three main cell types. Firstly, there are large cells with vesicular nuclei and prominent nucleoli, whose cytoplasm contains Nissl's substance. Secondly, there are numerous cells with ballooned nuclei which contain glycogen, stainable even in paraffin sections by Best's method, and by PAS, and removable by previous diastase treatment; and finally, there are many small cells of the glial type, with compact darkly staining nuclei (Fig. 2).

The central region contains numerous slit-like cavities, lined with ependyma and surrounded by trabeculae of pale neuro-epithelial cells. This structure is reminiscent of the pars nervosa of the hypophysis (Fig. 3).

Apart from this, most of the neural tissue is composed of the three cell types already described, with considerable areas showing a characteristic grouping of glial and ependymal cells about small vessels and cavities, resulting in a distinctly pinealomatous appearance (Fig. 4). PTAH stained preparations show the ependymal cells particularly well, with their typical long polarized processes ending in branched tufts or swellings about a small vessel or cavity (Fig. 5). Frozen sections stained by Bielchowsky's method, reveal the presence of large numbers of neuro-fibrils (Fig. 6, 7).

---

EXPLANATION OF PLATES

FIG. 1.—Diagram of structure of the bisected retro-ocular tumour. ×8·5.

FIG. 2.—Neurons containing Nissl's granules, glial cells, and vacuolated nuclei containing glycogen. (From Region A in the diagram.) Einarsen's stain. ×360.

FIG. 3.—Trabeculated neuro-epithelial region (Region B) showing ependymal-lined cavities. Note resemblance to *pars nervosa* tissue. H. and E. ×90.

FIG. 4.—Detail of pinealomatous region. Note the radiate arrangement of ependymal cells, and the condensation of dark staining glial cells. (Region anterior to B.) ×280.

FIG. 5.—Ependymal cells, showing polarisation of their processes and, bottom right, a terminal tuft to a process. (Region posterior to B.) Frozen section, Bielchowsky. × 1150.

FIG. 6.—Oil-immersion photograph of a frozen section from the pinealomatous region, showing a pear-shaped neuron, and the aggregation of glial cells and ependymal processes about an out-of-focus capillary. (Region anterior to A.) Bielchowsky. ×1150.

FIG. 7.—Another region of the pinealoma, showing diversity of cell types and the abundance of fibrils. (Region anterior to A.) Frozen section, Bielchowsky. ×900.

FIG. 8.—Mucus-secreting intestinal epithelium lining a main cavity in the glandular portion. There is a perfect sub-mucous coat and muscularis mucosae. (Region between A and C). Trichrome. ×90.

FIG. 9.—One of the two villiform structures extending into the major lumen of the glandular part. (Region C.) Note absence of muscle, vascularity and the modified investing epithelium (Region C.) An epithelial cyst, resembling a feather follicle, is at left centre. H. and E. ×21.

FIG. 10.—Base of the second " villus ", showing collagenous and elastic stroma, and the transition of the epithelium near the top to a type resembling corneal-scleral junction epithelium as it continues down. (Region between A and C.) ×90.

FIG. 11.—Lymphoid tissue, a group of glands resembling embryonic bronchial epithelium, and a capillary network resembling embryo lung. (Region A.) H. and E. ×115.

FIG. 12.—" Embryonic lung tissue ", associated with glandular epithelium and cartilage. (Region D.) H. and E. ×140.

FIG. 13.—A field containing, from left to right, fat, a small focus of epithelial cells, connective tissue, osteoid, bone, and possible bronchial epithelium. (Region posterior to D.) H. and E. ×90.

KEY TO FIGURE 1

1. Pinealomatous tissue.

2. Plain muscle.

3. Connective tissue.

4. Fat.

5. Cartilage.

6. Blood vessels.

7. " Lung ".

8. Intestinal epithelium.

9. Bone.

10. Striated muscle.

1

Campbell.

2

3

4

5

6

7

8

9

10

11

12

13

*The glandular epithelium*

Nearly all the cavities except those in the neural part and a few small slit-like spaces, presumably lymph channels, are lined with a single layer of epithelial cells. These vary from a flattened type investing the aforementioned villi, to a tall pale columnar mucus-secreting type lining the cavities and the proximal part of the glandular evaginations arising therefrom (Fig. 8). Not all of these cells are simultaneously active mucus secretors, as thionin blue and PAS stained material shows. The nuclei are basal and are very dense and crescent shaped. Small isolated glands are often associated with duct-like structures, which may be either simple, or much branched, and are usually not actively secreting mucus. The transition between active and resting cells is abrupt. The two prominent villiform processes which project into the lumen of the largest cavity are covered with an epithelium continuous with the mucus-secreting lining (Fig. 9) but showing a transition near the villus base to cells which resemble conjunctival epithelium at the corneal-palpebral junction (Fig. 10) and which later become flattened as the free tip of the villus is approached. The core of the villi is composed of vascular connective tissue. Glandular epithelium wherever situated is associated with a connective tissue submucous layer, external to which are thin inner and thick outer circular coats of plain muscle interspersed with connective tissue. Small discrete clusters of pale nuclei resembling the cells of the myenteric plexus lie in a thin fibrous matrix between the muscle coats.

*Respiratory epithelium*

A few minute cysts and tubular glands are lined with several layers of basophilic cells resembling embryonic bronchial epithelium. These cells do not appear to be ciliated, although typical basal bodies are present near the free border. The cysts are, for the most part, in the vicinity of the cartilage plaques and the presumptive embryonic lung, and they are usually associated with lymphoid tissue. The " lung " varies from a loose network of epithelial tissue, to an open plexus of thin-walled tubes, some of which contain a few red blood cells. Although the interpretation of these tubes is open to doubt, owing to the variable structure, it is thought not unreasonable to suggest tentatively a resemblance to lung capillaries (Fig. 11, 12).

*Muscle components*

As already mentioned, the plain muscle is confined to the posterior and dorsal two-thirds of the tumour, and is mainly arranged in a circular manner around the glandular cavities. Small isolated bundles of cells also occur scattered in a haphazard manner throughout the connective tissue stroma.

The striated muscle is present as a fairly broad band external to the cartilage, bone and " respiratory epithelium " (Fig. 13), and is otherwise unremarkable.

*The connective tissue*

Apart from the specialised glial fibres in the neural portion, the connective tissue is confined to the capsule, which is not intact at the anterior portion, and to the remainder of the tumour, where it forms a submucous layer of the stroma in general. It is mainly of an undifferentiated embryonic type with plump pale nuclei and abundant cytoplasm, but showing little mitotic activity. That investing the neural part resembles meningeal tissue.

*Lymphoid tissue*

Small widely scattered foci of lymphoid tissue occur in association with glands or glandular epithelium. A few aggregations are also present in the neural part of the tumour. The majority of the cells are mature lymphocytes, the remainder being plasma cells and reticulum cells.

*Epithelial cyst*

The small cyst visible in Fig. 9 is composed of cuboidal ectodermal cells, containing numerous minute brownish-black cytoplasmic granules showing a distinct aggregation at the free border. The lumen of the cyst contains a plug of dead epithelial cells. There is thus some resemblance to a feather follicle.

### DISCUSSION

The pineal body or epiphysis cerebri is represented in the vertebrates from the cyclostomes upwards. In the lamprey (Petromyzon) it shows the greatest degree of organization, being a double structure, consisting of parapineal (parietal organ) and pineal, both of which approach the dorsal surface of the skull and form well defined eye-cups at the distal extremities. At their proximal connection with the diencephalon, they are definitely glandular. In the selachians, the glandular structure is predominant and only the parapineal forms an eye-cup. In teleosts the eye-cup is lost, but they show a marked glandular structure of pineal and parapineal, both of which apparently secrete into the cerebro-spinal fluid. In amphibia the pineal, and in reptiles the parietal organ is modified for light reception, and in Sphenodon, the parietal organ is a particularly well developed eye-cup. Fossil saurian skulls have a dorsal foramen, which presumably housed the pineal or " third eye ", and this is still present in the parietal bones of many lizards. In *Rana temporaria*, the pineal is visible beneath the skin in the interocular region as the organ of Stieda. Histologically this is a degenerate organ, being an irregular mass of epithelial cells some of which resemble retinal rods. There is evidence, however, that in this animal the pineal organ still functions as a light receptor (Heerd and Dodt, 1961).

The only member of the class Aves in which the development of the pineal has been studied in any detail is the domestic fowl, and here Lillie (1952) states that it is first discernible as an evagination from the roof of the diencephalon, at about the 30–35 somite stage (approximately 60 hours). It subsequently grows out as a long narrow tube, dilated distally and giving off numerous hollow buds. There is no parietal organ. In the adult fowl the pineal body is situated behind the thalami as a small body, triangular in section and attached to the roof of the third ventricle. Its capsule is provided by the pia mater, which extends into the organ to form trabeculae surrounding hollow intercommunicating cords of neuro-epithelial cells. The majority of these are glial cells resembling astrocytes, but the lumen of the cord is lined with ependymal cells. The general appearance is gland-like, although no morphological evidence of secretion can be seen, and physiological evidence of secretion in birds and other animals is inconclusive. Thus Foà (1914) found that pinealectomy had no effect on female chicks, but retarded the growth of male chicks until they were 2–3 months old, when they became normal in weight. Kozelka (1932) found no effects in chicks after implantation experiments, but Shellabarger (1952) found that pinealectomy inhibited testis growth in young chicks, and that this could be restored to normal by the injection of lyophilized beef pineal. Lowenstein (1952), while showing that extracts of pineal were not physiologically inert, remarked on the fact that those workers who have obtained positive experimental evidence of activity in chicks are agreed that this activity is confined to males, It is perhaps not without significance that pineal teratomas are practically confined to the human male, and that " about one half of the cases show precocious sexual and bodily development " (Bochner and Scarff, 1938).

The present tumour almost certainly arose from pineal tissue. The possibility of a hypophyseal origin is remote, in view of the extreme rarity of tumours of the pars nervosa in man, and the total absence of reports in lower animals. Older reports of teratomas in the human pituitary are not now accepted, the view being that they were craniopharyngiomas showing variable metaplasia (Saphir, 1959). Infiltration of the posterior lobe of the human hypophysis with pinealomatous cells has been recorded by Walton (1949), who also discusses the so-called " ectopic or parapineal teratomas ", and concludes that the evidence for their pineal origin is inconclusive. Although there was no opportunity in the present case to examine the cranial cavity and its contents, the information provided showed that there were no nervous symptoms, i.e. that the growth was probably almost entirely confined to the orbit, resulting in a severe proptosis. This being so, we are faced with the problem of how it gained access to the orbital space.

There appear to be two alternative ways of spread from the pineal region, one being forward between the cerebral hemispheres, down and back to the optic nerves, and so to the optic foramen in the hinder end of the interorbital septum ; while an alternative route would seem to be laterally between the cerebral hemisphere and optic lobe to the optic tracts and the foramen. Unfortunately there is no direct evidence for either. It is possible that an intra-cranial tumour grew as thin subarachnoid strands of tissue along one or other of these routes, to expand as soon as the orbital cavity was reached. Alternatively it may have arisen *de novo* from an orbital locus of developmentally displaced pluripotential cells. At first sight there is no reason why a group of such cells should not form pinealomatous tissue amongst other tissue. It seems more likely, however, that the tumour arose from an intracranial site ; the literature on human pineal teratomas records that these very rare tumours frequently have a layer of either normal or adenomatous pineal tissue in juxtaposition (Frankl-Hochwart, 1909; Getzeit, 1896, cited by Ewing, 1942). Willis (1958) states that in humans, almost all intracranial teratomas arise in the vicinity of the pineal gland, quadrigeminal plate or walls of the posterior part of the third ventricle, including the pituitary region. In those human cases where an intra-cranial pinealoma was found together with a normal pineal gland, Ewing (1942) suggested that the tumour may have arisen from other primitive outpocketings from the diencephalon, e.g. the pre-commisural organs of the mid-habenular corpusculum parietale. No reference to a retro-ocular teratoma associated with pinealomatous tissue in man could be found, although about 20 human orbital teratomas have been reported during the last 100 years. Burnier and Salles (1945), quoted by Harbert (1949), collected 17 of these references, and see also Kamal (1954) and Winter (1960). Although a strictly orbital origin cannot be excluded in the present case, the association with pinealomatous tissue, not recorded in teratomas similarly sited in man, and the known propensity of teratomas to arise in or near the mid-line of the embryonic axis, weighs against such a site of origin.

There is no doubt that the present tumour is a true teratoma. All the primitive germ layers are present. The ectoderm is represented by the epithelial cyst, and by ependymal cells and nerve cells which in different parts bear a structural resemblance to the pars nervosa of the hypophysis, and to pinealomatous tissue. The mesodermal derivatives include cartilage, bone, muscle, fat, etc., and the entodermal components are mucus secreting alimentary epithelium, and possibly respiratory epithelium. Moreover, the tumour shows a marked capacity to produce organoids, as witness the structure which, despite the absence of a squamous lining to the mucous membrane, resembles oesophagus, even to the presence of groups of myenteric nerve cells. The identification of lung tissue is more tentative, but the proximity of cartilage, bone, and cells resembling embryonic respiratory epithelium, albeit non-ciliated, may be significant.

Very little information as to the significance of intra-nuclear glycogen appears in the literature. Cameron (1952) states that it is present in a proportion of liver nuclei, especially in diabetes in the human subject, but there is no indication of its occurrence in nerve cell nuclei. Ewing (1942) mentions glycogen in cytoplasmic vacuoles in ependymal neurogliomas, and quotes Marburg (1909) who traced the origin of such tumours from pineal parenchyma. Two other tissues associated with C.N.S. which could feasibly contain glycogen would be the notochord and its neoplastic counterpart, the chordoma, and secondly the glycogen body, which is an ovoid mass of glycogen-rich cells of unknown function lying in the sinus rhomboideus between the spinal lumbo-sacral dorsal funiculi of the fowl. However, while not dismissing the possibility that notochord cells may be present in this tumour, the general histology of the neural part bears no resemblance either to reported cases of human chordoma, or to the glycogen body.

Of all the tumours that the oncologist, whether experimental or diagnostic, encounters, the rare teratoma, with its apparent (although deceptive) attempts to produce a soma within a soma, is surely the most fascinating. Many pathologists in their daily routine cannot help occasionally losing sight of the fact that the tissues with which they deal arose and differentiated from two cells in a manner which is still largely not understood. Weismannian views die hard, and histologists and pathologists may thus find themselves occasionally surprised at the way in which tissues regarded by them as mature and fixed can form other histologically distinct tissues, even though these are always of the same germ-layer origin. Tissue culture studies, and observations on de-differentiation of cells in reparative processes should by now have done much to dispel the notions of the fixity of " differentiated " cells, while the more exotic forms of metaplasia underline the ability of cells to become profoundly modified in response to anatomical or physiological need. A further instance of the labile properties of tissues is afforded by the ovarian stroma, which can give rise to a variety of at first sight unrelated neoplasms. All of these examples serve to indicate that the somatic cell retains at least some of the multipotentiality which exists in cells in early embryonic life.

Thus, in post-embryonic life, cell differentiation results in a modification of morphology and function, but does not, save in rare instances, produce cells normally derived from a different primitive germ layer ; whereas in the early embryo at the primitive streak stage, any cell in any of the three germ layers is capable of differentiating into any tissue or organ.

It is this fact which has led to the hypothesis that teratomas arise from tissue primordia which have somehow become physiologically segregated in early embryonic life, thus escaping the influence of the dorsal lip primary organiser in the blastopore at the stage of gastrulation. (In the avian embryo, the primitive streak corresponds to an elongated blastopore.) The earlier this segregation takes place, the greater the capacity for self-differentiation of the particular blastoderm cells involved. These, in extreme cases, may possess all the potentialities of the fertilized ovum, and consequently, in the presence of organizers, but no individuation field, expresses them in an organoid but otherwise completely chaotic manner (see Needham, 1950, for discussion). This is the view accepted by Willis (1958) in preference to the numerous alternative hypotheses, and in this he is in accord with Nicholson (1950). Certainly it is the only theory of the origin of teratomas which satisfactorily explains the absence of organisation and their marked tendency to occur in pre-axial median or closely paramedian sites.

### SUMMARY

A unique case is recorded of a retro-ocular teratoma associated with pinealomatous tissue, which occurred in a six-week old male " broiler " chick. All three primitive germ layers are represented, and a variety of tissues and histioid structures are described in some detail. Our knowledge of the origin and possible function of the pineal in vertebrates is briefly discussed, as also is the significance of tumours arising therefrom.

I am indebted to Dr. W. P. Blount, F.R.C.V.S., and to Mr. D. A. P. Grattan, M.R.C.V.S., of British Oil and Cake Mills Ltd., for sending the specimen and for subsequent information regarding the post-mortem findings. All expenses for this investigation were borne by the British Empire Cancer Campaign.

### REFERENCES

Bagg, J. J.—(1936) *Amer. J. Cancer*, **26**, 69.
Bochner, S. J. and Scarff, J. E.—(1938) *Arch. Surg. Lond.*, **36**, 303.
Burnier, P. and Salles, M.—(1945) *Arg. Inst. Perido Burnier.*, **7**, 114. (Quoted by Harbert, 1949.)
Cameron, G. R.—(1952) ' Pathology of the Cell '. Edinburgh (Oliver and Boyd).
Campbell, J. G.—(1951) *Brit. J. Cancer*, **5**, 69.
Carleton, R. L., Friedman, N. B. and Bomze, E. J.—(1953) *Cancer*, **6**, 464.
Ewing, J.—(1942) ' Neoplastic Diseases '. London (W. B. Saunders Co.).
Falin, L. I.—(1941) *Z. mikr.-anat. Forsch.*, **49**, 193.
*Idem* and Anissimowa, W. M.—(1940) *Z. Krebsforsch.*, **50**, 339.
Foà, C.—(1914) *Arch. ital. biol.*, **61**, 79.
Frankl-Hochwart—(1909) } Cited by Ewing, J.—(1942). ' Neoplastic Diseases '.
Getzeit, I. D.—(1896) } London (W. B. Saunders Co.).
Harbert, F.—(1949) *Arch. Ophthal., N.Y.*, **42**, 451.
Heerd, E. and Dodt, E.—(1961) *Pflüg. Arch. ges. Physiol.*, **274**, 33.
Kamal, A.—(1954) *Bull. Soc. ophthal. Égypte*, **47**, 195.
Kozelka, A. W.—(1932) *Proc. Soc. exp. Biol. N.Y.*, **30**, 842.
Lillie, F. R.—(1952) ' Development of the Chick '. New York (Henry Holt & Co.).
Lowenstein, M. G.—(1952) *Exp. Med. Surg.*, **10**, 135.

MARBURG, O.—(1909) *Arb. neurol. Inst. (Inst. Anat. Physiol. ZentNerv.) Univ. Wien*, **17**, 217. (Cited by Ewing, 1942.)
MARGULIÉS, A.—(1901) *Neurol. Zbl.*, **20**, 1026.
MASHAR, U.—(1932) *Virchows Arch.*, **285**, 155.
MICHALOWSKY, I.—(1926) *Zbl. allg. Path. path. Anat.*, **38**, 585.
NEEDHAM, J.—(1950) 'Biochemistry and Morphogenesis'. London (Cambridge University Press).
NICHOLSON, G. W. DE P.—(1950) 'Studies on Tumour Formation'. London (Butterworth & Co.).
SAPHIR, O.—(1959) 'A Text of Systemic Pathology'. Vol. II. Grune and Stratton.
SCHLOTTHAUER, C. F. AND KERNOHAN, J. W.—(1935) *Amer. J. Cancer*, **24**, 350.
SHELLABARGER, C. J.—(1952) *Endocrinology*, **51**, 152.
SHIMA, R.—(1908) *Neurol. Zbl.*, **27**, 889.
WALTON, K.—(1949) *J. Path. Bact.*, **61**, 11.
WILLIS, R. A.—(1958) 'The Borderland of Embryology and Pathology'. London (Butterworth & Co.).—(1960) 'The Pathology of Tumours'. 3rd Edition. London (Butterworth & Co.).
WINTER, F. C.—(1960) *Arch. Ophthal., N.Y.*, **64**, 163.